# A Facile Preparation and Energetic Characteristics of the Core/Shell CoFe_2_O_4_/Al Nanowires Thermite Film

**DOI:** 10.3390/mi11050516

**Published:** 2020-05-20

**Authors:** Chunpei Yu, Wei Ren, Ganggang Wu, Wenchao Zhang, Bin Hu, Debin Ni, Zilong Zheng, Kefeng Ma, Jiahai Ye, Chenguang Zhu

**Affiliations:** 1 School of Chemical Engineering, Nanjing University of Science and Technology, Nanjing 210094, China; yuchunpei@njust.edu.cn (C.Y.); hallowg@163.com (G.W.); hubinnjust@163.com (B.H.); zilongzh@yeah.net (Z.Z.); makefeng@njust.edu.cn (K.M.); yejiahai@njust.edu.cn (J.Y.); zcg_lnkz@163.com (C.Z.); 2 Shaanxi Applied Physics and Chemistry Research Institute, Xi’an 710061, China; nidebin@hust.edu.cn; 3 State Key Laboratory for Manufacturing Systems Engineering, Xi’an Jiaotong University, Xi’an 710049, China

**Keywords:** nanoenergetic materials, nanothermite, composite metal oxide, nanowires structure, core/shell, energy release

## Abstract

In this study, CoFe_2_O_4_ is selected for the first time to synthesize CoFe_2_O_4_/Al nanothermite films via an integration of nano-Al with CoFe_2_O_4_ nanowires (NWs), which can be prepared through a facile hydrothermal-annealing route. The resulting nanothermite film demonstrates a homogeneous structure and an intense contact between the Al and CoFe_2_O_4_ NWs at the nanoscale. In addition, both thermal analysis and laser ignition test reveal the superb energetic performances of the prepared CoFe_2_O_4_/Al NWs nanothermite film. Within different thicknesses of nano-Al for the CoFe_2_O_4_/Al NWs nanothermite films investigated here, the maximum heat output has reached as great as 2100 J·g^−1^ at the optimal thickness of 400 nm for deposited Al. Moreover, the fabrication strategy for CoFe_2_O_4_/Al NWs is also easy and suitable for diverse thermite systems based upon other composite metal oxides, such as MnCo_2_O_4_ and NiCo_2_O_4_. Importantly, this method has the featured advantages of simple operation and compatibility with microsystems, both of which may further facilitate potential applications for functional energetic chips.

## 1. Introduction

Nanothermites are generally composed of a metal fuel (mostly Al) and an oxidizer (mostly metal oxides and fluoropolymers) [1,2,3,4,5,6,7,8]. At least one component in the nanothermites should be on the nano scale. Most probably benefitted from their enhanced contact area and reduced mass diffusion distance between fuel and oxidant, nanothermites exhibit satisfactory performances in energy release, reaction rate and ignition delay time, when compared with those of bulk or micro counterparts [9,10,11,12,13]. In addition, their ignition and energy release properties can be tuned by regulating the sizes and shapes, interface contact and stoichiometric ratios of these two components. Therefore, nanothermites are strongly desired in the application fields of microelectromechanical systems (MEMS), propulsion, thermal batteries, gas generators, ignition, initiation and so on [14,15,16,17,18,19].

The properties of energetic thermites largely depend on both a meticulous design of nano-structure and an intense assembly between fuels and oxidizers. Inspired by these strategies, a variety of nanostructured thermites, e.g. porous nanothermite films [20], core/shell structured thermites [21,22], multilayered and sandwich-structure nanothermites [23,24,25,26], have been prepared. Among these reported nanostructured thermites, the core/shell nanothermite film has been widely investigated as a one-dimensional array architecture [27,28,29,30]. Such an architecture can lead to great improvements in the interfacial contact, intimacy of reactive components, and nanoscale mixing uniformity, and thus potentially boosting its energetic performances [31]. Although CuO/Al [32], Co_3_O_4_/Al [33] and NiCo_2_O_4_/Al [34] core/shell nanowire (NW) structures have demonstrated notable advantages in not only outstanding reactivity but also a significantly low activation energy. However, few investigations have been made on the core/shell nanothermite films involving other species of NWs structured metal oxides and composite metal oxides in particular. Therefore, it is highly desirable to develop different NW structured thermites for the demand of diversity in nanothermites.

**Figure 1:**
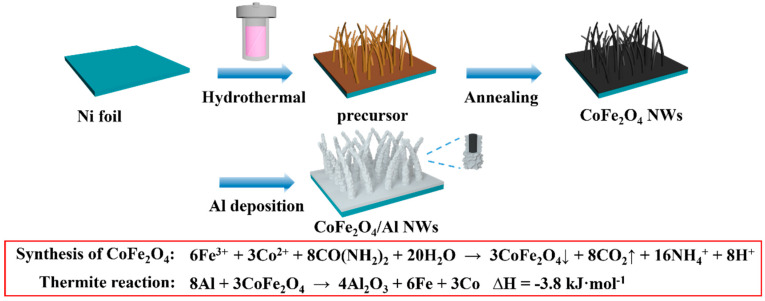
Schematic diagram for the fabrication of the core/shell CoFe_2_O_4_/Al nanothermite film.

Here, we demonstrate, by the example of CoFe_2_O_4_ NWs as a template, that the core/shell composite metal oxide/Al NWs nanothermite film can be effectively achieved. As schematically illustrated in Figure 1, the CoFe_2_O_4_ NWs array film has been firstly fabricated on the surface of a Ni substrate by means of a simple hydrothermal and annealing process and then a following deposition of nano Al through controllable magnetron sputtering to realize the core/shell CoFe_2_O_4_/Al NWs nanothermite film. The distinct NWs arrays structure can result in great improvements in the distribution and contact degree between fuel and oxidizer for an enhanced energy release and ease of ignition. An adjustment in the thickness of the nano-Al deposition can effectively lead to a control in both the molar ratio of fuel/oxidizer and energy release properties of the prepared nanothermite film. Moreover, the prepared CoFe_2_O_4_/Al NWs nanothermite film can be easily integrated into MEMS to achieve functional energetic chips. In addition, such an approach proposed here can also be applicable to the construction of various composite metal oxides/Al NWs, such as MnCo_2_O_4_/Al, NiCo_2_O_4_/Al and so on.

## 2. Materials and Methods

### 2.1. Materials

Fe(NO_3_)_3_·9H_2_O, Co(NO_3_)_2_·6H_2_O, CO(NH_2_)_2_, NH_4_F, Mn(SO_4_)_2_·H_2_O, Ni(NO_3_)_2_·6H_2_O and anhydrous ethanol were purchased from the Kelong Chemical Reagent Company (Chengdu, China). Ni foil and acetone were obtained from the Sinopharm Chemical Reagent Company (Shanghai, China). All chemicals were of analytical grade to be used as received without any further purification. For all experiments, deionized water (Milli-Q) was used.

### 2.2. Synthesis of the CoFe_2_O_4_ NWs Film

In the typical synthesis, a piece of Ni foil was cleaned ultrasonically first with acetone for 10 min and washed subsequently with deionized water and ethanol several times. In general, 2 mmol Co(NO_3_)_2_·6H_2_O and 4 mmol Fe(NO_3_)_3_·9H_2_O were dissolved in 60 mL deionized water under vigorous stirring to obtain a homogeneous precursor solution. The cleaned Ni foil was transferred into a 100 mL Teflon-lined stainless autoclave containing the precursor solution. The autoclave was sealed and maintained at 120 °C for 5 h. After reaction, the Ni substrate was taken out and washed with DI water and ethanol several times, followed by drying at 60 °C and then annealing in N_2_ at 400 °C for 5 h to obtain the CoFe_2_O_4_ NWs. For the synthesis of MnCo_2_O_4_ NWs or NiCo_2_O_4_ NWs, the processes were carried out in a similar way. The molar ratio of Co(NO_3_)_2_·6H_2_O and Mn(SO_4_)_2_·H_2_O was 2:1. The molar ratio of Co(NO_3_)_2_·6H_2_O and Ni(NO_3_)_2_·6H_2_O was 2:1.

### 2.3. Synthesis of the CoFe_2_O_4_/Al NWs Nanothermite Film

Nano-Al film was deposited over the CoFe_2_O_4_ NWs film by magnetron sputtering to realize the core/shell CoFe_2_O_4_/Al NWs nanothermite film. For all the magnetron sputtering processes, Ar gas with purity 99.99% was used as the working gas, with a flow rate of 50 sccm. Both the target holder and the substrate holder were cooled by circulating cooling water to make sure that the working temperature was under 25 °C. The Al layer thicknesses analyzed in this paper were set to be 200, 400, and 600 nm for a flat surface, respectively.

### 2.4. Characterizations

The structural and compositional information of the prepared materials were obtained by X-ray diffraction (XRD), (Bruker D8 Advance, Bruker, Karlsruhe, Germany). The morphological features and the element distribution of the obtained samples on Ni substrates were characterized by field emission scanning electron microscopy (SEM) (Quanta 250F, FEI, Hillsboro, OR, USA), equipped with an energy dispersive X-ray spectrometer (EDS), transmission electron microscopy (TEM) (FEI Tecnai G2 20 LaB6, FEI, Hillsboro, OR, USA), and high resolution transmission electron microscopy (HRTEM) (FEI Tecnai G2 F30 S-Twin, FEI, Hillsboro, OR, USA). The element mappings were also performed on the HRTEM. Differential scanning calorimetry (DSC) (TGA/DSC 3+, Mettler Toledo, Zurich, Switzerland), was used to determine the reaction heats of the nanothermites from 300 to 900 °C at a heating rate of 10 °C·min^−1^ under 30 mL·min^−1^ N_2_ flow. In addition, the ignition performances of the nanothermite film were studied by a Nd:YAG laser device and a high-speed camera. The wavelength, the pulse width and the incident laser energy were 1064 nm, 6.5 ns and 30 mJ, respectively.

## 3. Results and Discussion

With our starting chemical materials, the formation mechanism of the CoFe_2_O_4_ phase can be described in the following two steps. (1) Precursor NWs can be grown on the Ni substrate by a facile hydrothermal method with the assistance of F^−^ [35,36]. Typically, Co^2+^ and Fe^3+^ ions are fully coordinated with F^−^ to form [CoF_x_]^(x−2)−^ and [FeF_y_]^(y−3)−^ in the as-prepared precursor solution, respectively. As the temperature of the hydrothermal reaction ramps up to 120 °C, urea generates a large amount of CO_3_^2−^ and OH^−^ ion, both of which can slowly displace F^−^ in the [CoF_x_]^(x−2) −^ and [FeF_y_]^(y−3)−^ to result in the formation of a nucleus. As the reaction time increases, numerous nuclei are aligned to a wire-like structure; finally the process continues until formation of the Co-Fe-precursor NWs arrays are grown directly on the Ni substrate. (2) After annealing at 400 °C for 5 h in N_2_, the Co-Fe-precursor NWs gradually decompose to develop CoFe_2_O_4_ NWs. The chemical reactions responsible for the formation of CoFe_2_O_4_ NWs are presented in the following formulas:

Co^2+^ + xF^−^ → [CoF_x_]^(x-2)-^, (1)

Fe^2+^ + yF^−^ → [FeF_y_]^(y-3)-^, (2)

H_2_NCONH_2_ + H_2_O → 2NH_3_ + CO_2_, (3)

CO_2_ + H_2_O → CO_3_^2-^ + 2H^+^, (4)

NH_3_·H_2_O → NH_4_^+^ + OH^−^, (5)

[CoF_x_]^(x-2)-^ + 2[FeF_y_]^(y-3)-^ + 0.5(2-z) CO_3_^2-^ + (6+z)OH^−^ +nH_2_O → Co(OH)_z_(CO_3_)_0.5(2-z)_·2Fe(OH)_3_·nH_2_O + (x+2y)F^−^ (6)

Co(OH)_z_(CO_3_)_0.5(2-z)_·2Fe(OH)_3_·nH_2_O → CoFe_2_O_4_ + (1−0.5z)CO_2_ +(n+3+0.5z)H_2_O. (7)

**Figure 2:**
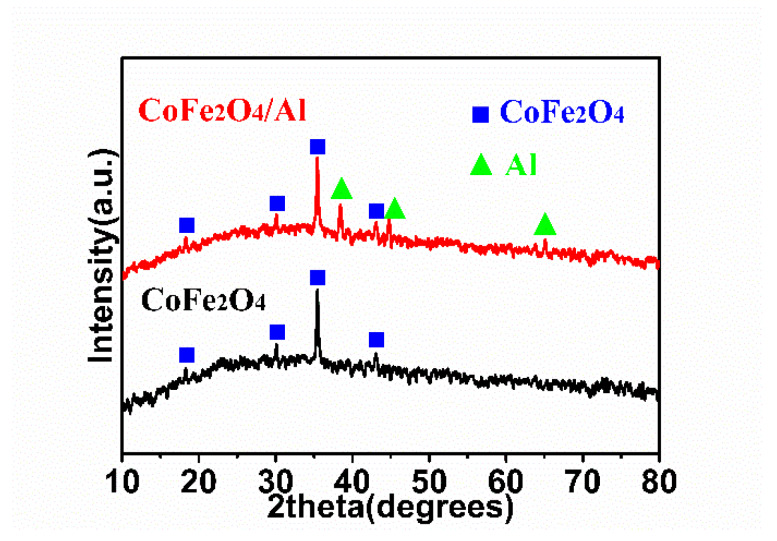
The XRD patterns of the CoFe_2_O_4_ nanowires (NWs) film and the CoFe_2_O_4_/Al NWs nanothermite film.

The phase composition and crystal structure of the CoFe_2_O_4_ film and CoFe_2_O_4_/Al nanothermite film are studied using XRD measurements, as shown in Figure 2. It should be pointed out here that the samples are scraped off the Ni substrate before XRD analysis. All the detected diffraction peaks from the CoFe_2_O_4_ film at 18.3°, 30.1°, 35.5° and 43.1° (labelled with blue squares) can be assigned to the (111), (220), (311) and (440) planes of CoFe_2_O_4_ cubic spinel phase (JCPDS Card 22-1086), respectively. With respect to CoFe_2_O_4_/Al, it can be clearly observed that the three additional characteristic peaks at 38.5°, 44.7° and 65.1°, which are marked with green triangles, can be indexed as the (111), (200) and (311) planes of Al, respectively. All these evidences are consistent with the successful formation of the CoFe_2_O_4_/Al nanothermite films on the substrate surfaces. In addition, no other peaks are detected for any impurity so that there is little or even no reaction between oxidizer and Al during the magnetron sputtering process.

**Figure 3:**
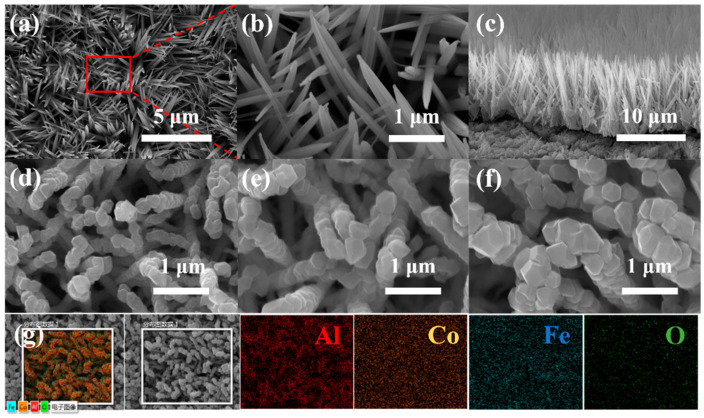
The scanning electron microscopy (SEM) images of the (**a**,**b**) CoFe_2_O_4_ NWs film, (**c**) CoFe_2_O_4_ NWs film from the side view, (**d**) CoFe_2_O_4_/Al NWs nanothermite film (Al = 200 nm), (**e**) CoFe_2_O_4_/Al NWs nanothermite film (Al = 400 nm), (**f**) CoFe_2_O_4_/Al NWs nanothermite film (Al = 600 nm) and (**g**) elemental mappings of CoFe_2_O_4_/Al NWs nanothermite film (Al = 400 nm).

The morphologies of CoFe_2_O_4_ NWs film and CoFe_2_O_4_/Al NWs nanothermite film are presented with different Al deposition thicknesses of 200, 400 and 600 nm, respectively, in Figure 3. The dense and compact CoFe_2_O_4_ NWs, whose average diameter is ca. 180 nm, are uniformly grown on the substrate surface, as shown in Figure 3a,b. Interestingly, the tip parts of the CoFe_2_O_4_ NWs structure are thinner than the root parts. As seen from the cross-section SEM image of the CoFe_2_O_4_ NWs (Figure 3c), NWs appear like numerous orderly-arranged grasses with ca. 8 μm in length to be rooted in the substrate. There is averagely a 2 μm layer of CoFe_2_O_4_ in thickness beneath the NWs structure, which contributes to the adhesion of the NWs structure to the substrate.

The SEM images are shown in Figure 3d–f for CoFe_2_O_4_/Al NWs nanothermite films with different thicknesses of Al deposition. The Al nanolayer can be effectively coated on the CoFe_2_O_4_ NWs to achieve core/shell CoFe_2_O_4_/Al NWs. In addition, the CoFe_2_O_4_/Al NWs still remain in the array structure without a change during the course of the Al coating. Furthermore, it is obvious that the surface of CoFe_2_O_4_/Al NWs becomes rough. The different deposition rates of Al on the sidewall of a single NWs could result in some nano-Al agglomerations to form a layer of granular coatings. Distinctly, the average diameter of the CoFe_2_O_4_/Al NWs expands with increasing thickness of Al deposition. The CoFe_2_O_4_/Al NWs can reach up to ca. 250 nm and 330 nm in average diameter for 200 nm and 400 nm thickness of the deposited Al, respectively (Figure 3d,e). However, it is worth noting that the deposited Al gradually aggregates on the top of the NWs until the NWs get fused with each other when the deposition thickness is 600 nm (Figure 3f), which prevents a further penetration of the Al atoms during magnetron sputtering to reduce the mixing uniformity between fuel and metal oxide. Moreover, Figure 3g shows the corresponding EDS elemental mappings of CoFe_2_O_4_/Al NWs nanothermite film at the Al deposition thickness of 400 nm. It can be observed that the Al, Co, Fe and O are uniformly distributed in this nanothermite film. As a result, it reveals that Al material is uniformly coated onto the CoFe_2_O_4_ NWs. 

The molar ratios between Al to CoFe_2_O_4_ are 2.00, 4.34 and 6.72 in the CoFe_2_O_4_/Al nanothermite film with the 200, 400 and 600 nm thicknesses of the Al deposition, respectively. The fuel/oxidizer molar ratio obviously rises with increasing thickness of the Al deposition. In addition, the molar ratio between Al to CoFe_2_O_4_ (4.34), which goes beyond the theoretical value (2.67) in the CoFe_2_O_4_/Al film with the 400 nm thickness of the Al deposition, reveals a slight surplus of fuel. It is worth noting that this proposed approach can be applied to fabricate various composite-metal-oxides-based thermite systems, such as MnCo_2_O_4_/Al (see Figures S1–S3, in Supplementary Materials) and NiCo_2_O_4_/Al (see Figures S4–S6, in Supplementary Materials).

**Figure 4:**
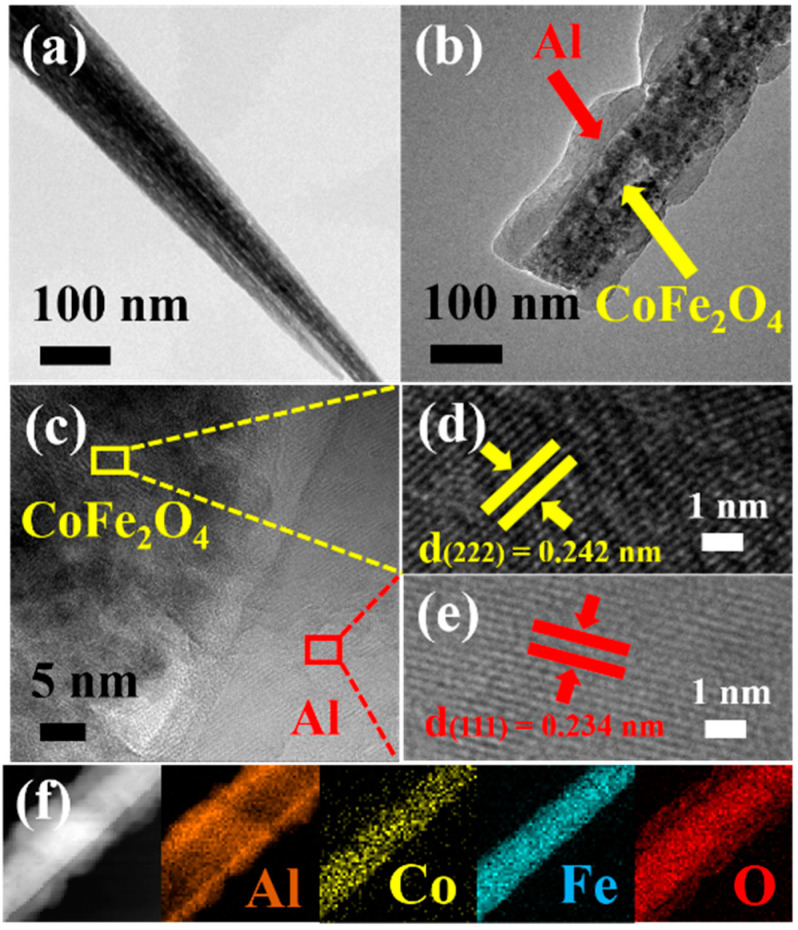
The transmission electron microscopy (TEM) images of the (**a**) CoFe_2_O_4_ NWs, (**b**) CoFe_2_O_4_/Al NWs (Al = 200 nm), (**c**,**d**,**e**) high resolution transmission electron microscopy (HRTEM) images of CoFe_2_O_4_/Al NWs (Al = 200 nm), and (**f**) the corresponding element mappings of CoFe_2_O_4_/Al NWs.

Both TEM and HRTEM were performed to further characterize the morphology and detailed structure of the CoFe_2_O_4_ NWs and CoFe_2_O_4_/Al NWs, as shown in Figure 4. The TEM image (Figure 4a) shows that each NW consists of many nanocrystallites. From the TEM image of Figure 4b, it can be found that the CoFe_2_O_4_ NWs is wrapped with a layer of nano-Al for ca. 35 nm averagely in thickness to form a core/shell structure CoFe_2_O_4_/Al NWs, which is well consistent with its SEM observations (Figure 3d). Meanwhile, the HRTEM characterizations of CoFe_2_O_4_/Al NWs reveal the intense contact between the Al and CoFe_2_O_4_ at the nanoscale (Figure 4c). In addition, the HRTEM image of the CoFe_2_O_4_ core (Figure 4d) displays clear lattice fringes with an interplanar spacing of 0.242 nm, which corresponds to the (222) crystal plane of the CoFe_2_O_4_. For the Al shell, lattice fringe with spacing value of 0.234 nm, as indicated in Figure 4e, corresponds to the (111) plane of the Al. Furthermore, the corresponding elemental mapping images (Figure 4f) for a single CoFe_2_O_4_/Al NW obviously reveals the core/shell structure for well-distributed Co, Fe and O elements in the core as well as Al in the shell. In addition, the O signal on the shell surface is derived from the inevitable oxidation of the nano-Al. Therefore, it can be definitely concluded that the core/shell nanothermite films are successfully fabricated in structure with CoFe_2_O_4_ NWs as a core and Al as a shell on the Ni substrate.

**Figure 5:**
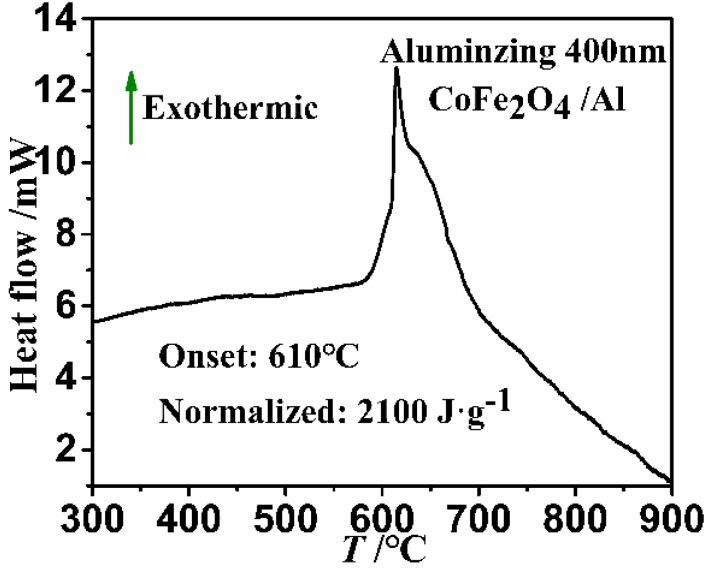
The differential scanning calorimetry (DSC) curve of the CoFe_2_O_4_/Al NWs nanothermite film (Al = 400 nm).

To acquire the preferable energy release of CoFe_2_O_4_/Al NWs nanothermite films, the different thicknesses of 200, 400 and 600 nm are tested for the Al deposition on a flat surface, respectively. It should be pointed out here that the CoFe_2_O_4_/Al NWs nanothermite films are scratched from the Ni substrate before DSC analysis. As shown in Figure 5, the DSC curve has been analyzed for CoFe_2_O_4_/Al NWs nanothermite film with the deposition thickness of 400 nm. It is clearly observed that only one sharp and large exothermic peak appears with an onset temperature of ca. 610 °C. The peak temperature at 615 °C demonstrates that the CoFe_2_O_4_/Al film has reacted prior to the melting point of Al (660 °C). In addition, the total released heat for the CoFe_2_O_4_/Al NWs nanothermite film is 2100 J·g^−1^, revealing a rapid redox reaction between CoFe_2_O_4_ and Al. Of note, it is the largest reaction heat among the investigated samples within different deposition thicknesses of Al (Figure S7, in Supplementary Materials). The data of exothermic performances are summarized in Table 1 for the samples with different thicknesses of Al. The energy release rises with increasing Al deposition from 200 to 400 nm in thickness, since an increment in the thickness of deposited Al layer will lead to an increase in overall fuel/oxidizer ratio of Al/CoFe_2_O_4_ [37], as confirmed by the EDS analysis. Nevertheless, the released heat starts to decrease when the deposited Al is too thick (greater than 600 nm here). Excess Al will reduce the oxidizer/fuel ratio. In addition, the maximum reaction heat is lower than the theoretical value. It could be attributed to not only the oxidation of the nano-Al layer but also a nonstoichiometric ratio for a complete reaction between Al and CoFe_2_O_4_ in the CoFe_2_O_4_/Al nanothermite film [27]. Despite all these, the energy release for CoFe_2_O_4_/Al NWs investigated in this work is larger than that of reported thermites, such as assembly Fe_2_O_3_/Al (2088 J·g^−1^) [38], sol gel Fe_2_O_3_/Al (1686 J·g^−1^) [38], physical mixing Fe_2_O_3_/Al (1097 J·g^−1^) [38] and porous Co_3_O_4_/Al (1740 J·g^−1^) [39].

The enhanced released heat of the CoFe_2_O_4_/Al NWs nanothermite film could be ascribed to the following reasons. Firstly, the obtained CoFe_2_O_4_/Al NWs are of excellent spatial uniformity. The CoFe_2_O_4_/Al film could be regarded as a compact integration of numerous individual core/shell NWs nanothermites at the nanoscale. Secondly, a low temperature and high vacuum environment during magnetron sputtering process can efficiently avoid the introduction of impurities and the potential reaction between Al and CoFe_2_O_4_. Thirdly, the synergistic effect of the composite metal oxide may promote the exothermic performance of the nanothermite to some extent, but the mechanism deserves further study. 

**Figure 6:**
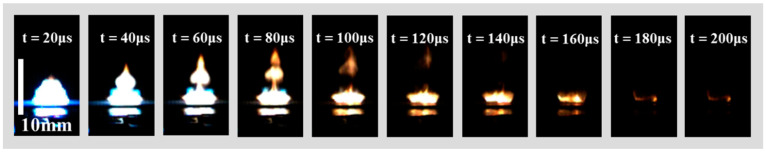
The high-speed camera photos of the ignition process of the CoFe_2_O_4_/Al NWs nanothermite film (Al = 400 nm).

The ignition performances of the prepared CoFe_2_O_4_/Al NWs nanothermite film are investigated using a laser ignition source and a high speed camera. The combustion process was recorded in Figure 6, where the interval time for each image frame is 20 μs. A very bright flash originated from the intense combustion of CoFe_2_O_4_/Al NWs nanothermite film is clearly visible, which is quite beneficial to the potential applications in micro-ignition and micro-actuation. The whole ignition duration lasts ca. 200 μs, which is significantly longer than that of the three-dimensionally ordered NiFe_2_O_4_/Al nanothermite film [40]. In addition, the flame height for the CoFe_2_O_4_/Al nanothermite film can reach as high as 10 mm. It reveals that this thermite film can be used as an ideal ignition material.

## 4. Conclusions

In conclusion, a novel core/shell CoFe_2_O_4_/Al NWs nanothermite film is fabricated via a facile hydrothermal process combined with a post annealing treatment and controllable magnetron sputtering. An adjustment in the deposition thicknesses of the Al shell is a qualitative way to realize the optimal fuel/oxidizer ratio of the nanothermite film. The thermodynamic results demonstrate the excellent energetic capability of the CoFe_2_O_4_/Al NWs nanothermite film (2100 J·g^−1^), which is much better than those of single metal oxide based thermite systems. In addition, a preliminary laser ignition test indicates that the as-prepared CoFe_2_O_4_/Al NWs nanothermite film can be successfully ignited. It is believed that the core/shell CoFe_2_O_4_/Al NWs nanothermite film prepared in this work will contribute to the studies of nanostructured energetic composite, especially for thermite systems based upon composite metal oxides. Finally, excellent energetic performance, easy synthesis process and high compatibility with MEMS technology meet the demands for functional energetic chips.

## Figures and Tables

**Table 1:** Comparisons of exothermic performances of the CoFe_2_O_4_/Al, Fe_2_O_3_/Al and Co_3_O_4_/Al.

Samples	Thickness for Deposited Al on CoFe_2_O_4_ This Work (nm)			Assembly Fe_2_O_3_/Al [36]	Sol-Gel Fe_2_O_3_/Al [36]	Physical Mixing-Fe_2_O_3_/Al [36]	Porous Co_3_O_4_/Al [37]
	200	400	600				
Heat of reaction (J·g^−1^)	1200	2100	1680	2088	1686	1097	1740

## Supplementary Materials

The following are available online at https://www.mdpi.com/2072-666X/11/5/516/s1, Figure S1: The SEM images of the MnCo_2_O_4_ NWs film, Figure S2: The SEM images of the MnCo_2_O_4_/Al NWs nanothermite film. (Al = 200 nm), Figure S3: The XRD pattern of the (a) MnCo_2_O_4_ NWs film and (b) MnCo_2_O_4_/Al NWs nanothermite film, Figure S4: The SEM images of the NiCo_2_O_4_ NWs film, Figure S5: The XRD pattern of the NiCo_2_O_4_ NWs film, Figure S6: The SEM images of the NiCo_2_O_4_/Al NWs nanothermite film. (Al = 200 nm), Figure S7: The DSC curve of CoFe_2_O_4_/Al NWs nanothermite film with different Al deposition thicknesses (a) 200 nm and (b) 600 nm.
