# Supplementary material for: A Facile Preparation and Energetic Characteristics of the Core/Shell CoFe2O4/Al Nanowires Thermite Film
Source: Micromachines (Basel). 2020 May 20;11(5):516. doi: 10.3390/mi11050516 (PMC7281481; doi:10.3390/mi11050516)
Supplement: Supplementary file 1 [file micromachines-11-00516-s001.pdf]

micromachines

MDPI

Supplementary Material: A Facile Preparation and Energetic Characteristics of the Core/Shell $CoFe_2O_4$/Al Nanowires Thermite Film

Chunpei Yu [1], Wei Ren [2,3,*], Ganggang Wu [1], Wenchao Zhang [1,*], Bin Hu [1], Debin Ni [2], Zilong Zheng [1], Kefeng Ma [1], Jiahai Ye [1] and Chenguang Zhu [1]

1 School of Chemical Engineering, Nanjing University of Science and Technology, Nanjing 210094, China; yuchunpei@njust.edu.cn (C.Y.); hallowg@163.com (G.W.); hubinnjust@163.com (B.H.); zilongzh@yeah.net (Z.Z.); makefeng@njust.edu.cn (K.M.); yejiahai@njust.edu.cn (J.Y.); zcg_lnkz@163.com (C.Z.)

2 Shanxi Applied Physics and Chemistry Research Institute, Xi'an 710061, China; nidebin@hust.edu.cn (D.N.)

3 State Key Laboratory for Manufacturing Systems Engineering, Xi'an Jiaotong University, Xi'an 710049, China.

* Correspondence: zhangwenchao@njust.edu.cn (W.Z.); rw0192@163.com (W.R.)

Received: 31 March 2020; Accepted: 8 May 2020; Published: date

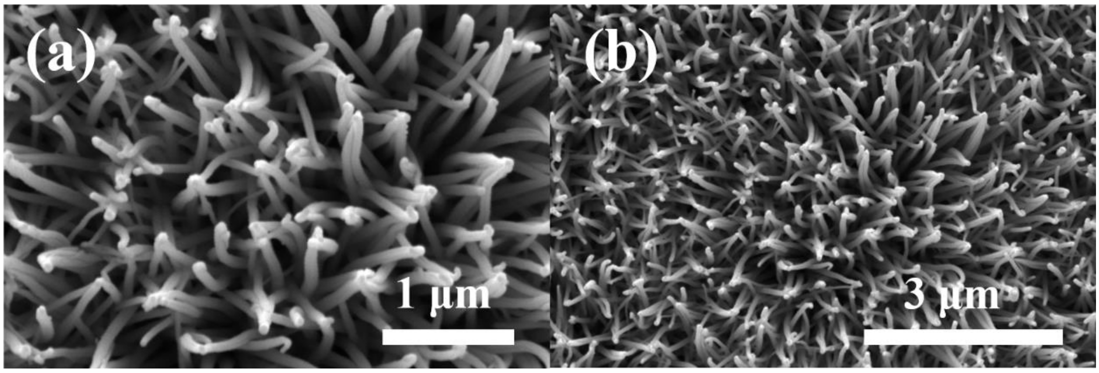

Figure S1. The SEM images of the $MnCo_2O_4$ NWs.

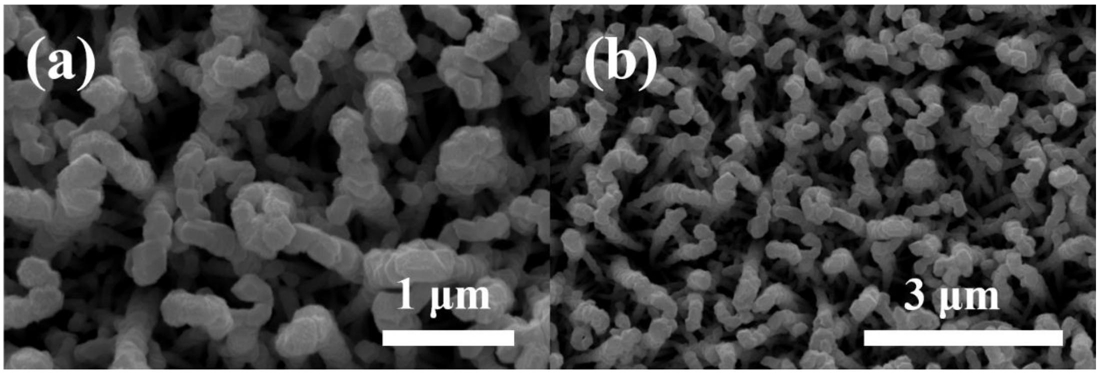

Figure S2. The SEM images of the $MnCo_2O_4$/Al NWs. (Al = 200 nm).

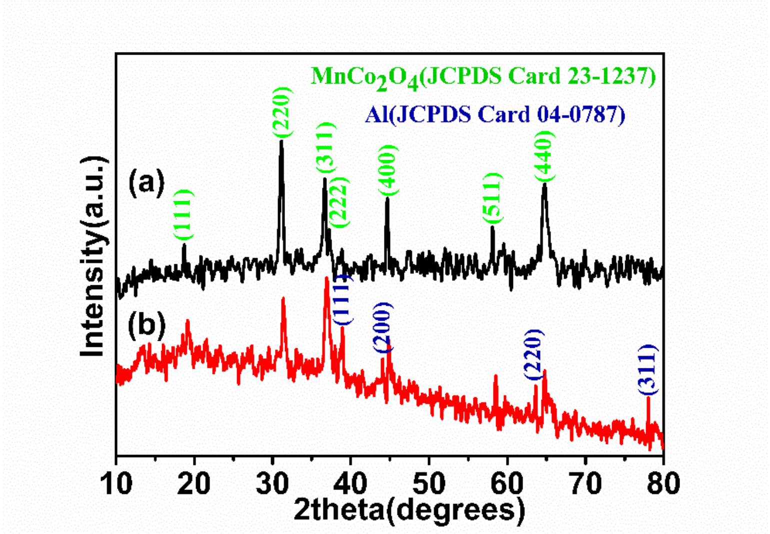

Figure S3. The XRD pattern of the (**a**) $MnCo_2O_4$ NWs and (**b**) $MnCo_2O_4$/Al NWs.

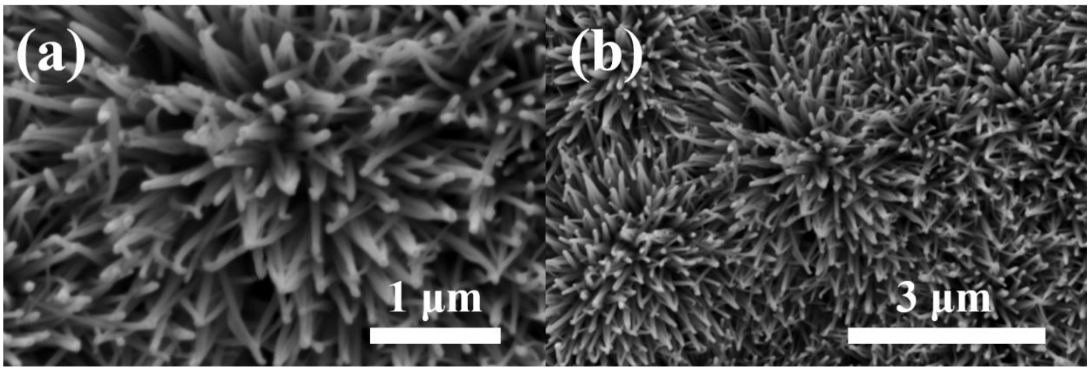

Figure S4. The SEM images of the $NiCo_2O_4$ NWs.

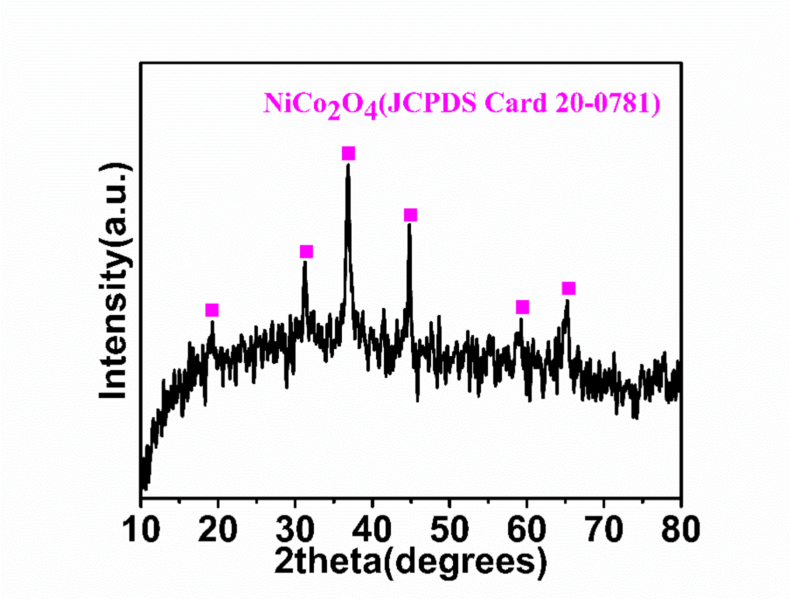

Figure S5. The XRD pattern of the $NiCo_2O_4$ NWs.

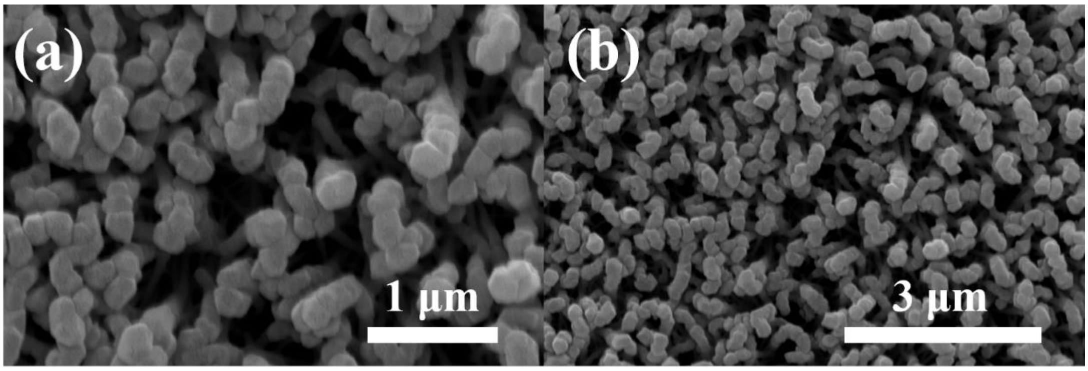

Figure S6. The SEM images of the $NiCo_2O_4$/Al NWs. (Al = 200 nm).

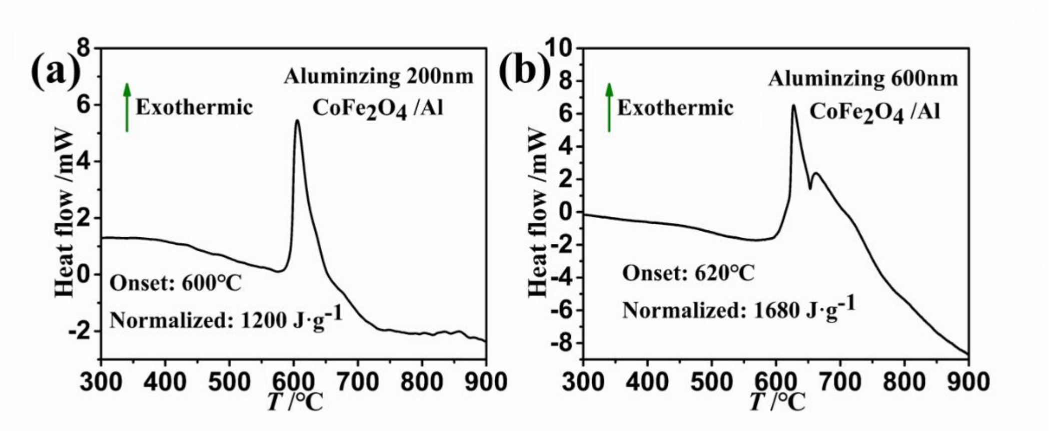

Figure S7. The DSC curve of $CoFe_2O_4$/Al with different Al deposition thicknesses (**a**) 200 nm and (**b**) 600 nm.
